# A straightforward approach towards combined α-amino and α-hydroxy acids based on Passerini reactions

**DOI:** 10.3762/bjoc.7.151

**Published:** 2011-09-19

**Authors:** Ameer F Zahoor, Sarah Thies, Uli Kazmaier

**Affiliations:** 1 Institute for Organic Chemistry, Saarland University, P.O. Box 151150, 66041 Saarbrücken, Germany

**Keywords:** amino acids, chelated enolates, epoxides, Passerini reactions, Ugi reactions

## Abstract

Complex amino acids with an α-acyloxycarbonyl functionality in the side chain are easily available through epoxide opening by chelated enolates and subsequent oxidation/Passerini reaction. This protocol works with both, aldehyde and ketone intermediates, as long as the ketones are activated by electron-withdrawing groups. In principle Ugi reactions are also possible, allowing the generation of diamino acid derivatives.

## Introduction

Multicomponent reactions (MCR) are a very popular and powerful tool in modern organic synthesis [1–4]. Besides a wide range of heterocycle syntheses [5] and catalytic cross coupling reactions [6], the isonitrile-based MCRs (IMCR) especially have developed exceptionally well during the last few decades [7–8]. Based on the pioneering work of Passerini, who observed the first three-component coupling of carbonyls with carboxylic acids and isonitriles in 1921 [9], the so-called Passerini reaction became a powerful tool for the synthesis of acylated α-hydroxyacid amides [10]. Later on, in 1961, Ugi and Steinbrückner reported the extension of this protocol by incorporating also a primary amine as a fourth component [11]. Therefore, the Ugi reaction is even more flexible than the Passerini approach, but both reactions together have made the IMCR highly popular in combinatorial chemistry [7–8].

Our group has been involved in amino acid and peptide synthesis for nearly two decades [12–13], and multicomponent reactions are known to play a dominant role [14–15]. In particular, the Ugi reaction has so far been used for the construction of exotic peptides [16–19] and cyclopeptides [20–21]. Herein we describe a straightforward protocol towards combined α-amino and α-hydroxy acids through Passerini reactions. Suitable amino acid precursors with an oxygen functionality in the side chain can be obtained by chelated enolate Claisen rearrangement [22–23] or transition metal-catalyzed allylic alkylation of chelated enolates [24] and subsequent oxidative cleavage of the γ–δ-unsaturated amino acids obtained.

## Results and Discussion

An alternative approach is based on regioselective ring opening of epoxides, followed by oxidation of the hydroxy amino acid formed. While aryl-substituted epoxides react preferentially at the benzylic position giving rise to the terminal primary alcohols [25], the corresponding alkyl-substituted epoxides provide secondary alcohols **1** by nucleophilic attack of the enolate at the sterically least-hindered position [26]. These alcohols can easily be oxidized by Swern-oxidation [27] or with Dess–Martin-periodinane (DMP) [28], giving rise to the required γ-oxo-amino acids **2** (Table 1). In principle both protocols are suitable for oxidation, but in general the yields obtained were better with DMP (82–93%), while under Swern conditions the yields were in the range of 75 ± 3%.

**Table 1:** Synthesis of γ-oxo-amino acids.

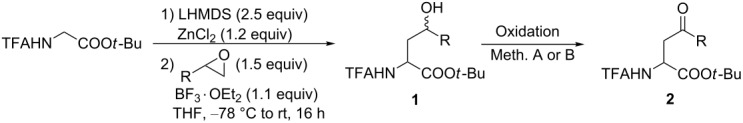						

Entry	**1**	R	Yield (%)	**2**	Yield (%)	
					Meth. A^a^	Meth. B^b^

1	**1a** [26]	CH_3_	92	**2a**	78	91
2	**1b** [26]	CH_2_Cl	82	**2b**	75	90
3	**1c** [26]	CH_2_OC_6_H_5_	86	**2c**	76	93
4	**1d**	CH_2_O-(*p*-Cl-C_6_H_4_)	88	**2d**	72	82
5	**1e**	CH_2_O-(*o*-NO_2_-C_6_H_4_)	84	**2e**	75	87
6	**1f**	CH_2_O-(*p*-NO_2_-C_6_H_4_)	83	**2f**	74	84

^a^Method A: Swern oxidation; ^b^Method B: DMP oxidation.

With these γ-oxo-α-amino acids **2** in hand, we investigated the Passerini reactions under neat conditions with acetic acid as the (liquid) acidic component and isocyano acetates as the reactive component (Table 2). Interestingly, no reaction was observed with the methyl-substituted oxo acid **2a** (entry 1); only the starting material was recovered. For this reason, we switched to activated ketones bearing an electron-withdrawing group at the α-position. With the chlorinated ketone **2b** the yield was 65% (entry 2), and similar results were obtained with a range of aryloxy-substituted derivatives **2c**–**2f** (entries 3–7). The new stereogenic center was formed without significant selectivity.

**Table 2:** Passerini reactions of γ-oxo-amino acids.

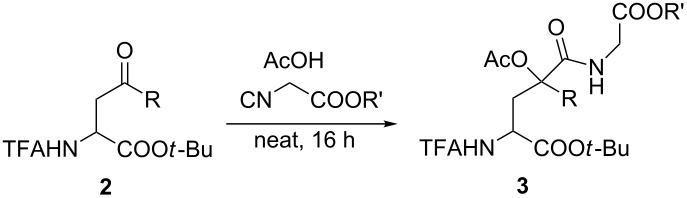					

Entry	**2**	R	R’	**3**	Yield (%)

1	**2a**	CH_3_	Me	**3a**	–
2	**2b**	CH_2_Cl	Me	**3b**	65
3	**2c**	CH_2_OC_6_H_5_	Me	**3c**	57
4	**2d**	CH_2_O-(*p*-Cl-C_6_H_4_)	Me	**3d**	69
5	**2e**	CH_2_O-(*o*-NO_2_-C_6_H_4_)	Me	**3e**	62
6	**2f**	CH_2_O-(*p*-NO_2_-C_6_H_4_)	Et	**3f**	69
7	**2d**	CH_2_O-(*p*-Cl-C_6_H_4_)	Et	**3g**	68

**Scheme 1:**
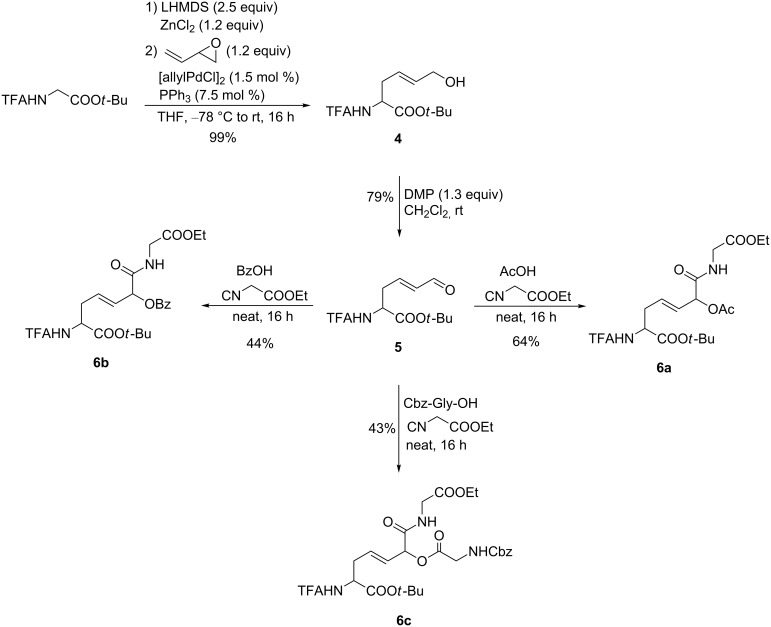
Passerini reactions of α,β-unsaturated aldehyde **5**.

To increase the synthetic potential of this protocol we also applied the Pd-catalyzed opening of a vinyl epoxide with our chelated enolate (Scheme 1) [29]. In this case an amino acid **4** with an allyl alcohol side chain was formed which could be oxidized to the α,β-unsaturated aldehyde **5**. Although these types of aldehydes are critical candidates in Passerini and Ugi reactions [30], we were interested to see if we could also obtain unsaturated Passerini adducts by this procedure. Our first attempts in CH_3_OH and CH_2_Cl_2_ were unsuccessful. While no reaction was observed in CH_2_Cl_2_, in CH_3_OH the only product (besides starting material) was the unsaturated acetal resulting from a nucleophilic attack of the solvent on the aldehyde group. Therefore, we decided to run the reaction also under neat conditions as reported for the γ-oxo-amino acids. With acetic acid as the acidic component the yield of **6a** was comparable to the previous examples. In principle, other acids such as benzoic acid or Cbz-protected glycine can be used as well. The lower yield obtained in these cases probably results from stirring problems under these solvent-free conditions.

**Scheme 2:**
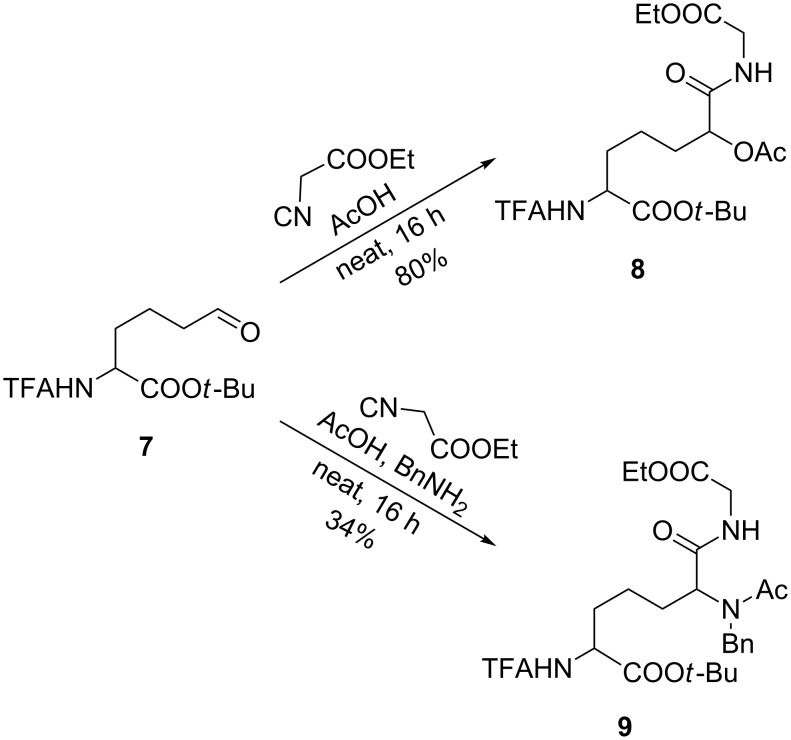
Passerini and Ugi reaction of saturated aldehyde **7**.

To circumvent the problems caused by the α,β-unsaturated aldehyde, we hydrogenated **4** before oxidation to obtain the saturated aldehyde **7**. And indeed, under our optimized reaction conditions the addition product **8** could be obtained in 80% yield (Scheme 2). In principle, Ugi reactions are also possible, as illustrated with the formation of **9**, although the yield was significantly lower in this case and the products are formed as a 1:1 diastereomeric mixture.

## Conclusion

In conclusion, we showed that the ring opening of epoxides, either directly or Pd-catalyzed, with chelated enolates combined with Passerini reactions is a suitable tool for the synthesis of highly functionalized α-hydroxy and α-amino acid derivatives. These new compounds are interesting building blocks for peptide-derived drugs. Attempts to improve the yields and to evaluate the scope and limitations are currently underway.

## Supporting Information

Supporting Information features detailed experimental procedures, NMR as well as analytical data of all compounds.

File 1 Experimental section.
