# Supplementary material for: A straightforward approach towards combined α-amino and α-hydroxy acids based on Passerini reactions
Source: Beilstein J Org Chem. 2011 Sep 19;7:1299–303. doi: 10.3762/bjoc.7.151 (PMC3182440; doi:10.3762/bjoc.7.151)
Supplement: File 1 — Experimental section. [file Beilstein_J_Org_Chem-07-1299-s001.pdf]

Supporting information

for

A straightforward approach towards combined α-amino and α-hydroxy acids based on Passerini reactions.

Ameer F. Zahoor, Sarah Thies and Uli Kazmaier*

Address: Institute for Organic Chemistry, Saarland University, P.O. Box 151150, 66041 Saarbrücken, Germany

Email: Uli Kazmaier - u.kazmaier@mx.uni-saarland.de

* Corresponding author

Experimental section

General Information

All reactions were carried out in oven-dried glassware (100 °C) under nitrogen atmosphere unless otherwise stated. Septa, disposable syringes and needles were used for the transfer of reagents and other liquid chemicals. For drying of organic phases water-free sodium sulfate was used.

^{1}H NMR-spectra were measured on a 400 MHz NMR spectrometer from Bruker (model AV-400). $CDCl_3$ was used as the solvent. The solvent peak was calibrated at 7.26 ppm. The analysis of spectra was performed with PC-software MestRe-C. The abbreviations used in the interpretation of NMR spectra are: s = singlet, d = doublet, dd = doublet of doublet, t = triplet, q = quartet, m = multiplet or br = broad. Chemical shifts are δ-values and were measured in ppm.

^{13}C NMR-spectra were also measured at a frequency of 100 MHz on a NMR spectrometer from Bruker (model AV-400). $CDCl_3$ was used as the solvent. The solvent peak was calibrated at 77.0 ppm. The analysis of spectra was done with PC-software MestRe-C. The abbreviations used for analysis are: s = singlet, d = doublet, t = triplet, q = quartet. Chemical shifts were δ-values and were measured in ppm.

Preparative flash column chromatography was performed through columns packed with silica gel grade 60 (35–70µm) purchased from Macherey-Nagel.

Melting points were measured in open glass capillaries on apparatus MEL-TEMP II purchased from Laboratory Devices and are uncorrected.

Thin-layer chromatography was performed with commercially available precoated Polygram® SIL-G/UV 254 plates purchased from Fluka. The detection of spots was achieved under UV-light, I_2-vapours or $KMnO_4$ solution.

High Pressure liquid chromatography was performed on the instrument purchased from Shimadzu (model 10A VP). As an achiral column, LiChrosorb Si-60 (250–4, diameter 5 µm) was used (Phenomenex). The evaluation was performed with Class VP-Software (Schimadzu).

Elemental analyses were performed at the Institute for Organic Chemistry, University of Saarland on the instrument Leco (model CHN900).

High resolution mass spectrometry (HRMS) was performed at the Institute for Organic Chemistry, University of Saarland on a MAT 95Q (Finnigan). The fragmentation was carried out through chemical ionization (CI) or electron ionization (EI).

Solvents were dried by refluxing the corresponding solvent over suitable drying agent. Tetrahydrofuran (THF) was dried over lithium aluminium hydride (LAH), dichloromethane (DCM) was dried over powdered CaH_2. Commercial grade solvents, such as ethyl acetate, hexane, and diethyl ether, were distilled prior to use.

***tert*-Butyl 2-(2,2,2-trifluoroacetamido)acetate (**TFA-Gly-O*t*-Bu)

In a three neck flask with an intensive cooler and cryostat, ammonia gas was condensed (~400 ml) at –78 °C. Under a nitrogen atmosphere *tert*-butyl bromoacetate (100 g, 0.51 mol) was added dropwise. The reaction mixture was stirred for 2 days at –78 °C and then it was warmed up to r.t. and the excess of ammonia was evaporated. The resulting solid was filtered off and was washed with diethyl ether. The filtrate was evaporated and the crude product was purified by bulb-to-bulb distillation (p = 10 torr, bp = 50 °C) giving rise to *tert*-butyl glycinate as a clear oil in 72% yield (48.6 g, 0.37 mol).

tert-Butyl glycinate (10.0 g, 76.2 mmol) was dissolved in methanol (250 ml). At 0 °C triethylamine (15.4 g, 153 mmol) was added, and finally methyl trifluoroacetate (21.7 g, 153 mmol) in methanol (100 ml) was added. The reaction mixture was allowed to warm up to r.t. overnight and then the methanol was evaporated. The resulting residue was dissolved in ethyl acetate and washed with water. The organic phase was dried (Na_2SO_4) and the solvent was evaporated. The crude product was

purified by bulb-to-bulb distillation (p = 3 torr, bp = 86 °C) and resulting in a clear oil in 84% yield (14.5 g, 64.0 mmol).

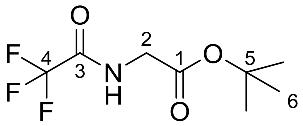

^{1}H NMR (400 MHz, $CDCl_3$): δ = 6.86 (s, 1H, NH), 4.01 (d, $^{3}J_{2,NH}$ = 4.9 Hz, 2H, 2-H), 1.49 (s, 9H, 6-H). **^{13}C NMR** (100 MHz, $CDCl_3$): δ = 167.3 (C-1), 156.6 ($J_{3,F}$ = 37.5 Hz, C-3), 116.6 ($J_{4,F}$ = 287 Hz, C-4), 83.6 (C-5), 42.0 (C-2), 27.9 (C-6).

**HRMS (ESI):**	Calculated	Found
$C_8H_{12}F_3NO_3$ $[M+1]^+$:	228.0803	228.0843

General procedure for ring opening reactions of epoxides[1]

In a Schlenk tube hexamethyldisilazane (497 mg, 3.08 mmol) was dissolved in dry THF (5.0 ml). The solution was cooled to –78 °C, and then a 1.6 M solution of *n*-BuLi (1.72 ml, 2.75 mmol) was added slowly. The solution was stirred for 10 min and the cooling bath was then removed and the solution was stirred for a further 10 min. In a second Schlenk flask $ZnCl_2$ (180 mg, 1.32 mmol) was dried with a heat gun under vacuum, and dissolved in THF (5.0 ml). The solution was cooled to room temperature, then Tfa-Gly-O*t*-Bu (250 mg, 1.1 mmol) was added and the mixture was cooled to –78 °C. The LHMDS solution was then added slowly. The resulting solution was stirred for 30 min at –78 °C. Then the corresponding epoxide (1.65 mmol) was added, followed by the addition of BF_3·OEt_2 (78.1 mg, 0.55 mmol). The reaction mixture was allowed to warm to r.t. overnight and then it was hydrolyzed with 1 M HCl and extracted thrice with ethyl acetate. The combined organic layers were dried (Na_2SO_4), the solvent was evaporated in vacuo, and the crude product was purified by flash chromatography (silica gel, hexanes/EtOAc).

***tert*-Butyl 4-hydroxy-2-(2,2,2-trifluoroacetamido)pentanoate (1a)**[1]

According to the general procedure for epoxide openings TFA-Gly-O*t*-Bu (250 mg, 1.1 mmol), propylene oxide (128 mg, 2.2 mmol), hexamethyldisilazane (497 mg, 3.08 mmol, 2.8 equiv), *n*-BuLi (1.72 ml, 2.75 mmol, 2.5 eq.) and BF_3·OEt_2 (172 mg, 1.2 mmol) were allowed to react to give **1a** after flash chromatography (silica, hexanes/EtOAc 8:2) in 92% yield (289 mg, 1.01 mmol) as a colorless oil. [TLC: DCM/Hex 95:5, R_f = 0.28].

[1] Kazmaier, U., Zahoor, A. F. *Arkivoc* **2011**, IV, 6–16.

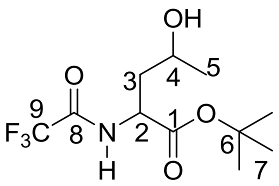

Major diastereomer:

^{1}H NMR (400 MHz, $CDCl_3$): δ = 7.66 (bs, 1 H, N-H), 4.59 (dt, $^3J_{2,3}$ = 8.1 Hz, $^3J_{2,NH}$ = 3.6 Hz, 1 H, 2-H), 3.86–3.89 (m, 1 H, 4-H), 2.74 (d, $^3J_{OH,4}$ = 3.2 Hz, 1 H, O-H), 1.92 (ddd, $^2J_{3a,3b}$ = 14.2 Hz, $^3J_{3a,4}$ = 10.4 Hz, $^3J_{3a,2}$ = 3.7 Hz, 1 H, 3a-H), 1.82 (ddd, $^2J_{3b,3a}$ = 14.3 Hz, $^3J_{3b,4}$ = 8.5 Hz, $^3J_{3b,4}$ = 2.6 Hz, 1 H, 3b-H), 1.48 (s, 9 H, 7-H), 1.26 (d, $^3J_{5,4}$ = 6.2 Hz, 3 H, 5-H). **^{13}C NMR** (100 MHz, $CDCl_3$): δ = 169.7, 157.4 (q, J = 37.2 Hz), 115.7 (q, J = 285.6 Hz), 83.2, 64.8, 51.5, 39.9, 27.8, 23.5.

Minor diastereomer (selected signals):

^{1}H NMR (400 MHz, $CDCl_3$): δ = 7.53 (bs, 1 H ,N-H), 4.42 (ddd, $^3J_{2,3}$ = $^3J_{2,H}$ = 6.2 Hz, 1 H, 2-H), 3.92–3.99 (m, 1 H, 4-H), 2.03 (ddd, $^2J_{3a,3b}$ = 14.5 Hz, $^3J_{3a,2}$ = 5.7 Hz, $^3J_{3a,4}$ = 3.2 Hz, 1 H, 3a-H), 1.96 (s, 1 H, O-H), 1.89 (ddd, $^2J_{3b,3a}$ = 14.5 Hz, $^3J_{3b,2}$ = 9.3 Hz, $^3J_{3b,4}$ = 6.5 Hz, 1 H, 3b-H), 1.45 (s, 9 H, 7-H), 1.23 (d, $^3J_{5,4}$ = 6.2 Hz, 3 H, 5-H). **^{13}C NMR** (100 MHz, $CDCl_3$): δ = 169.8, 156.8, (q, J = 37.3 Hz), 115.6 (q, J = 285.8 Hz), 83.1, 65.5, 52.1, 39.4, 27.8, 24.1.

**HRMS (CI):**	Calculated	Found
$C_{11}H_{18}F_3NO_4$ [M+1]$^+$:	286.1221	286.1273

Elemental Analysis:

$C_{11}H_{18}F_3NO_4$	Calculated	C 46.31	H 6.36	N 4.91
(285.26):	Found	C 46.46	H 6.21	N 5.18

***tert*-Butyl 5-chloro-4-hydroxy-2-(2,2,2-trifluoroacetamido)pentanoate (1b)[1]**

According to the general procedure for epoxide openings TFA-Gly-O*t*-Bu (250 mg, 1.1 mmol), epichlorohydrin (204 mg, 2.2 mmol), hexamethyldisilazane (497 mg, 3.08 mmol, 2.8 equiv), *n*-BuLi (1.72 ml, 2.75 mmol, 2.5 eq.) and $BF_3{\cdot}OEt_2$ (172 mg, 1.2 mmol) were allowed to react to give **1b** after flash chromatography (silica, hexanes/EtOAc 8:2) in 82% yield (288 mg, 0.90 mmol) as a colorless oil. [TLC: DCM/Hex 95:5, R_f = 0.28]

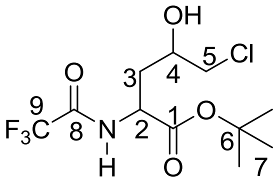

Major diastereomer:

^{1}H NMR (400 MHz, $CDCl_3$): δ = 7.70 (d, $^3J_{2,3}$ = 7.3 Hz, 1 H, N-H), 4.68 (dt, $^3J_{2,NH}$ = 7.7 Hz, $^3J_{2,3}$ = 3.8 Hz, 1 H, 2-H), 3.83–3.90 (m, 1 H, 4-H), 3.53 (dd, $^2J_{5a,5b}$ = 10.0 Hz, $^3J_{5a,4}$ = 4.0 Hz, 1 H, 5a-H), 3.49 (dd, $^2J_{5b,5a}$ = 10.0 Hz, $^3J_{5b,4}$ = 5.2 Hz, 1 H, 5b-H), 3.35 (d, $^3J_{OH,4}$ = 3.9 Hz, 1 H, O-H), 1.94–2.08 (m, 2 H, 3-H), 1.47 (s, 9 H, 7-H). **^{13}C NMR** (100 MHz, $CDCl_3$): δ = 169.2, 157.4 (q, *J* = 37.2 Hz), 115.7 (q, *J* = 285.6 Hz), 83.7, 66.7, 51.1, 48.7, 35.5, 27.8.

Minor diastereomer:

^{1}H NMR (400 MHz, $CDCl_3$): δ = 7.46 (d, $^3J_{2,3}$ = 6.0 Hz, 1 H, N-H), 4.50 (ddd, $^3J_{2,3}$ = $^3J_{2,H}$ = 5.9 Hz, 1 H, 2-H), 3.91–3.98 (m, 1 H, 4-H), 3.57 (dd, $^2J_{5a,5b}$ = 11.2 Hz, $^3J_{5a,4}$ = 4.1 Hz, 1 H, 5a-H), 3.49 (dd, $^2J_{5b,5a}$ = 11.2 Hz, $^3J_{5b,4}$ = 6.6 Hz, 1 H, 5b-H), 2.72 (d, $^3J_{OH,4}$ = 5.1 Hz, 1 H, O-H), 2.20 (ddd, $^2J_{3a,3b}$ = 14.5 Hz, $^3J_{3a,2}$ = 5.9 Hz, $^3J_{3a,4}$ = 2.9 Hz, 1 H, 3a-H), 1.98–2.06 (m, 1 H, 3b-H), 1.47 (s, 9 H, 7-H). **^{13}C NMR** (100 MHz, $CDCl_3$): δ = 169.4, 157.4 (q, *J* = 37.2 Hz), 115.7 (q, *J* = 285.6 Hz), 83.6, 68.4, 51.3, 49.3, 35.0, 27.7.

**HRMS (CI):**	Calculated	Found
$C_{11}H_{17}{}^{37}ClF_3NO_4$ $[M]^+$:	321.0763	321.0795

Elemental Analysis:

$C_{11}H_{17}ClF_3NO_4$	Calculated	C 41.32	H 5.36	N 4.38
(319.70):	Found	C 41.43	H 5.08	N 4.54

***tert*-Butyl 4-hydroxy-5-phenoxy-2-(2,2,2-trifluoroacetamido)pentanoate (1c)[1]**

According to the general procedure for epoxide openings TFA-Gly-O*t*-Bu (250 mg, 1.1 mmol), commercially available phenyl glycidyl ether (330 mg, 2.2 mmol), hexamethyldisilazane (497 mg, 3.08 mmol, 2.8 equiv), *n*-BuLi (1.72 ml, 2.75 mmol, 2.5 eq.) and $BF_3{\cdot}OEt_2$ (172 mg, 1.2 mmol) were allowed to react to give **1c** after flash chromatography (silica, hexanes/EtOAc 8:2) in 86% yield (357 mg, 0.95 mmol) as a colorless oil. [TLC: DCM/Hex 95:5, R_f = 0.34]

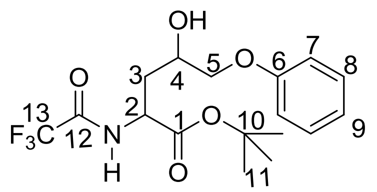

Major diastereomer:

^{1}H NMR (400 MHz, $CDCl_3$): δ = 7.77 (d, $^3J_{NH,2}$ = 7.8 Hz, 1 H, N-H), 7.23–7.29 (m, 2 H, 7-H), 6.95 (dd, $^3J_{9,8}$ = 7.4 Hz, 1 H, 9-H), 6.85–6.87 (m, 2 H, 8-H), 4.71 (dt, $^3J_{2,NH}$ = 7.5 Hz, $^3J_{2,3}$ = 4.4 Hz, 1 H, 2-H), 4.01–4.11 (m, 1 H, 4-H), 3.91 (dd, $^2J_{5a,5b}$ = 7.9 Hz, $^3J_{5a,4}$

= 3.1 Hz, 1 H, 5a-H), 3.88 (dd, $^{2}J_{5b,5a}$ = 7.8 Hz, $^{3}J_{5b,4}$ = 5.2 Hz, 1 H, 5b-H), 3.83 (d, $^{3}J_{OH,4}$ = 3.3 Hz, 1 H, O-H), 1.98–2.09 (m, 2 H, 3-H), 1.47 (s, 9 H, 11-H). **^{13}C NMR** (100 MHz, $CDCl_3$): δ = 169.2, 158.1, 157.4 (q, *J* = 37.4 Hz), 129.5, 121.4, 115.7 (q, *J* = 285.6 Hz), 114.4, 83.4, 71.3, 67.4, 51.2, 34.5, 27.9.

Minor diastereomer:

^{1}H NMR (400 MHz, $CDCl_3$): δ = 7.43 (bs, 1 H, N-H), 7.23–7.28 (m, 2 H, 7-H), 6.96 (dd, $^{3}J_{9,8}$ = 7.4 Hz, 1 H, 9-H), 6.85–6.87 (m, 2 H, 8-H), 4.51 (ddd, $^{3}J_{2,NH}$ = $^{3}J_{2,3a}$ = $^{3}J_{2,3b}$ = 5.9 Hz, 1 H, 2-H), 4.09–4.16 (m, 1 H, 4-H), 3.94 (dd, $^{2}J_{5a,5b}$ = 9.4 Hz, $^{3}J_{5a,4}$ = 3.5 Hz, 1 H, 5a-H), 3.88 (dd, $^{2}J_{5b,5a}$ = 9.3 Hz, $^{3}J_{5b,4}$ = 7.1 Hz, 1 H, 5b-H), 2.52 (d, $^{3}J_{OH,4}$ = 4.3 Hz, 1 H, O-H), 2.05–2.24 (m, 1 H, 3-H), 1.47 (s, 9 H, 11-H). **^{13}C NMR** (100 MHz, $CDCl_3$): δ = 169.4, 158.1, 157.4 (q, *J* = 37.4 Hz), 129.6, 121.4, 115.7 (q, *J* = 285.6 Hz), 114.5, 83.4, 71.4, 67.4, 51.7, 33.8, 27.8.

**HRMS (CI):**	Calculated	Found
$C_{17}H_{22}F_3NO_5$ $[M]^+$:	377.1450	377.1447

Elemental Analysis:

$C_{17}H_{22}F_3NO_5$	Calculated	C 54.11	H 5.88	N 3.71
(377.35):	Found	C 54.64	H 5.62	N 3.95

***tert*-Butyl 5-(4-chlorophenoxy)-4-hydroxy-2-(2,2,2-trifluoroacetamido)pentanoate (1d)**

According to the general procedure for epoxide openings TFA-Gly-O*t*-Bu (250 mg, 1.1 mmol), commercially available 4-chlorophenyl glycidyl ether (407 mg, 2.2 mmol), hexamethyldisilazane (497 mg, 3.08 mmol, 2.8 equiv), *n*-BuLi (1.72 ml, 2.75 mmol, 2.5 eq.) and $BF_3{\cdot}OEt_2$ (172 mg, 1.2 mmol) were allowed to react to give **1d** after flash chromatography (silica, hexanes/EtOAc 8:2) in 88% yield (399 mg, 0.97 mmol) as a colorless oil. [TLC: DCM/Hex 95:5, R_f = 0.32]

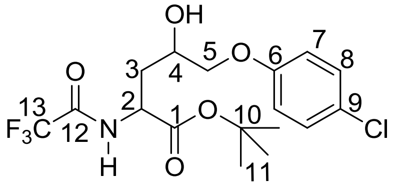

Major diastereomer:

^{1}H NMR (400 MHz, $CDCl_3$): δ = 7.77 (d, $^{3}J_{NH,2}$ = 7.0 Hz, 1 H ,N-H), 6.83-6.85 (m, 4 H, 7-H, 8-H), 4.73 (dt, $^{3}J_{2,NH}$ = 7.4 Hz, $^{3}J_{2,3}$ = 4.5 Hz, 1 H, 2-H), 4.06-4.14 (m, 1 H, 4-H), 3.79-3.93 (m, 2 H, 5-H), 3.11 (d, $^{3}J_{OH,4}$ = 3.3 Hz, 1 H, O-H), 2.03-2.13 (m, 2 H, 3-H), 1.49 (s, 9 H, 11-H). **^{13}C NMR** (100 MHz, $CDCl_3$): δ = 171.5, 157.4 (q, *J* = 37.4 Hz),

152.2, 130.0, 129.0, 115.7 (q, J = 285.6 Hz), 115.4, 114.9, 83.4, 72.6, 65.5, 55.8, 51.2, 27.9.

Minor diastereomer:

^{1}H NMR (400 MHz, $CDCl_3$): δ = 7.44 (s, 1 H, N-H), 4.53 (ddd, $^3J_{2,NH}$ = $^3J_{2,3a}$ = $^3J_{2,3b}$ = 5.9 Hz, 1 H, 2-H), 4.07-4.13 (m, 1 H, 4-H), 3.77 (s, 3 H, 10-H), 2.19-2.25 (m, 1 H, 3a-H), 1.49 (s, 9 H, 12-H). **^{13}C NMR** (100 MHz, $CDCl_3$): δ = 171.3, 152.1, 129.9, 129.0, 115.5, 114.7, 83.4, 72.6, 65.5, 55.6, 51.2, 27.8.

**HRMS (CI):**	Calculated	Found
$C_{17}H_{21}ClF_3NO_5$ $[M]^+$:	411.1060	411.1075

tert-Butyl 4-hydroxy-5-(2-nitrophenoxy)-2-(2,2,2-trifluoroacetamido)pentanoate (1e)

According to the general procedure for epoxide openings TFA-Gly-O*t*-Bu (250 mg, 1.1 mmol), commercially available 2-nitro phenyl glycidyl ether (429 mg, 2.2 mmol), hexamethyldisilazane (497 mg, 3.08 mmol, 2.8 equiv), *n*-BuLi (1.72 ml, 2.75 mmol, 2.5 eq.) and $BF_3{\cdot}OEt_2$ (172 mg, 1.2 mmol) were allowed to react to give **1e** after flash chromatography (silica, hexanes/EtOAc 8:2) in 84% yield (390 mg, 0.92 mmol) as a colorless oil. [TLC: DCM/Hex 95:5, R_f = 0.31]

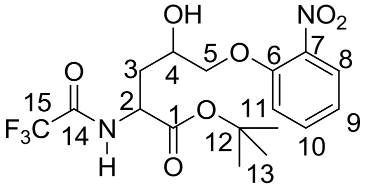

Major diastereomer:

^{1}H NMR (400 MHz, $CDCl_3$): δ = 7.87 (dd, $^3J_{8,9}$ = 8.5 Hz, $^3J_{8,10}$ = 1.7 Hz, 1 H, 8-H), 7.81 (d, $^3J_{NH,2}$ = 7.5 Hz, 1 H, N-H), 7.52–7.57 (m, 1 H, 11-H), 7.01–7.06 (m, 2 H, 9-H, 10-H), 4.53 (ddd, $^3J_{2,NH}$ = $^3J_{2,3}$ = 5.7 Hz, 1 H, 2-H), 4.13–4.18 (m, 2 H, 5-H), 4.01–4.05 (m, 1 H, 4-H), 2.91 (d, $^3J_{OH,4}$ = 4.9 Hz, 1 H, O-H), 2.04–2.16 (m, 2 H, 3-H), 1.49 (s, 9 H, 13-H). **^{13}C NMR** (100 MHz, $CDCl_3$): δ = 169.1, 157.4 (q, J = 37.4 Hz), 151.8, 139.8, 134.4, 125.9, 121.3, 115.7 (q, J = 285.6 Hz), 115.1, 83.5, 73.2, 67.1, 51.1, 34.2, 27.8.

Minor diastereomer:

^{1}H NMR (400 MHz, $CDCl_3$): δ = 7.87 (dd, $^3J_{8,9}$ = 8.1 Hz, $^3J_{8,10}$ = 1.7 Hz, 1 H, 8-H), 7.53–7.57 (m, 1 H, 11-H), 7.81 (d, $^3J_{NH,2}$ = 7.3 Hz, 1 H, N-H), 7.05–7.11 (m, 2 H, 9-H, 10-H), 4.72 (dt, $^3J_{2,NH}$ = 7.4 Hz, $^3J_{2,3}$ = 4.2 Hz, 1 H, 2-H), 4.11–4.18 (m, 2 H, 5-H), 4.00–4.05 (m, 1 H, 4-H), 3.52 (d, $^3J_{OH,4}$ = 2.5 Hz, 1 H, O-H), 2.30 (ddd, $^2J_{3a,3b}$ = 14.5 Hz, $^3J_{3a,2}$ = 5.6 Hz, $^3J_{3a,4}$ = 2.9 Hz, 1 H, 3a-H), 2.10 (ddd, $^2J_{3b,3a}$ = 14.6 Hz, $^3J_{3b,4}$ = 9.6

Hz, $^3J_{3b,2}$ = 5.4 Hz, 1 H, 3b-H), 1.51 (s, 9 H, 13-H). **^{13}C NMR** (100 MHz, $CDCl_3$): δ = 169.3, 151.9, 134.5, 126.0, 121.3, 115.0, 83.6, 73.2, 66.7, 51.4, 33.6, 27.8.

**HRMS (CI):**	Calculated	Found
$C_{17}H_{21}F_3N_2O_7$ $[M]^+$:	423.1334	423.1413

tert-Butyl 4-hydroxy-5-(4-nitrophenoxy)-2-(2,2,2-trifluoroacetamido)pentanoate (1f)

According to the general procedure for epoxide openings TFA-Gly-O*t*-Bu (250 mg, 1.1 mmol), commercially available 4-nitro phenyl glycidyl ether (429 mg, 2.2 mmol), hexamethyldisilazane (497 mg, 3.08 mmol, 2.8 equiv), *n*-BuLi (1.72 ml, 2.75 mmol, 2.5 eq.) and $BF_3 \cdot OEt_2$ (172 mg, 1.2 mmol) were allowed to react to give **1f** after flash chromatography (silica, hexanes/EtOAc 8:2) in 83% yield (386 mg, 0.91 mmol) as a colorless oil. [TLC: DCM/Hex 95:5, R_f = 0.31]

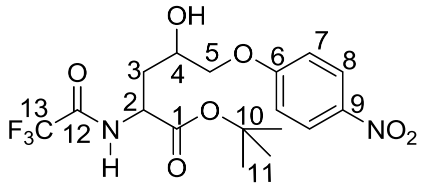

Major diastereomer:

^{1}H NMR (400 MHz, $CDCl_3$): δ = 7.81 (d, $^3J_{NH,2}$ = 7.5 Hz, 1 H, N-H), 7.08 (d, $^3J_{2,3}$ = 8.3 Hz, 2 H, 7-H), 6.77–6.80 (m, 2 H, 8-H), 4.73 (dt, $^3J_{2,NH}$ = 7.4 Hz, $^3J_{2,3}$ = 4.2 Hz, 1 H, 2-H), 4.06-4.16 (m, 1 H, 4-H), 3.81–3.97 (m, 2 H, 5-H), 2.03–2.13 (m, 2 H, 3-H), 1.49 (s, 9 H, 12-H). **^{13}C NMR** (100 MHz, $CDCl_3$): δ = 169.2, 157.4 (q, *J* = 37.4 Hz), 156.0, 130.0, 129.0, 115.7 (q, *J* = 285.6 Hz), 114.3, 113.9, 83.4, 71.6, 67.5, 51.2, 34.5, 27.9.

Minor diastereomer:

^{1}H NMR (400 MHz, $CDCl_3$): δ = 7.48 (d, $^3J_{NH,2}$ = 5.5 Hz, 1 H, N-H), 4.54 (ddd, $^3J_{2,NH}$ = $^3J_{2,3a}$ = $^3J_{2,3b}$ = 5.9 Hz, 1 H, 2-H), 2.03–2.13 (m, 2 H, 3-H), 1.49 (s, 9 H, 12-H). **^{13}C NMR** (100 MHz, $CDCl_3$): δ = 169.2, 155.8, 130.7, 83.3, 71.6, 67.4, 51.6, 33.8, 27.8.

**HRMS (CI):**	Calculated	Found
$C_{17}H_{21}F_3N_2O_7$ $[M]^+$:	423.1334	423.1416

General procedure for Dess–Martin oxidations

To a solution of the corresponding γ-hydroxy amino acid ester **1** or **4** (1.32 mmol) in dry dichloromethane, Dess–Martin periodinane (721 mg, 1.7 mmol) was added at 0 °C under nitrogen and the mixture was stirred at room temperature for 3 h. The reaction was quenched with a saturated solution of $NaHCO_3$ containing $Na_2S_2O_3$, and then the mixture was extracted with dichloromethane. The organic layers were

washed with a saturated solution of NaCl, dried over Na_2SO_4 and the solvent was removed in vacuo. The corresponding γ-keto amino acid ester was obtained after column chromatography (silica gel, EtOAc/hexane).

General procedure for Swern oxidations

To a solution of oxalyl chloride (67 mg, 0.52 mmol) in dry dichloromethane (0.5 ml) at –78 °C, DMSO (82.8 mg, 1.06 mmol) was added, the solution was stirred for 10 min, and then a solution of the corresponding γ-hydroxy amino acid ester **1** (0.26 mmol) was added dropwise. The reaction mixture was stirred at the same temperature for another 1 h and then triethylamine (134 mg, 1.32 mmol) was added. The cooling bath was removed after 15 min, and the reaction mixture was allowed to warm to room temperature. Water (5 ml) was added and the reaction mixture was stirred for an additional 10 min. The reaction mixture was extracted with dichloromethane, the organic layers were washed with 1 N HCl, saturated NaCl soln., dried over Na_2SO_4 and the solvent was removed in vacuo. The corresponding γ-keto amino acid ester was obtained after column chromatography (silica gel, EtOAc/ hexane).

***tert*-Butyl 4-oxo-2-(2,2,2-trifluoroacetamido)pentanoate (2a)**

According to the general procedure for Dess–Martin oxidations alcohol **1a** (377 mg, 1.32 mmol) and Dess–Martin periodinane (721 mg, 1.7 mmol) were reacted to give **2a** after flash chromatography (silica, hexanes/EtOAc 8:2) in 91% yield (340 mg, 1.20 mmol) as a colorless oil. [TLC: Hex/EA 75:25, R_f = 0.53].

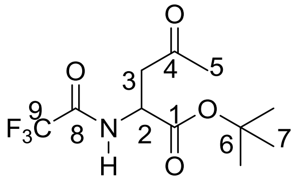

^{1}H NMR (400 MHz, $CDCl_3$): δ = 7.37 (bs, 1 H ,N-H), 4.59 (td, $^3J_{2,NH}$ = 7.8 Hz, $^3J_{2,3}$ = 3.8 Hz, 1 H, 2-H), 3.22 (dd, $^2J_{3a,3b}$ = 18.6 Hz, $^3J_{3a,2}$ = 4.0 Hz, 1 H, 3a-H) , 3.97 (dd, $^2J_{3b,3a}$ = 18.6 Hz, $^3J_{3b,2}$ = 4.0 Hz, 1 H, 3b-H), 2.10 (s, 3 H, 5-H), 1.43 (s, 9 H, 7-H). **^{13}C NMR** (100 MHz, $CDCl_3$): δ = 206.1, 168.0, 156.8 (q, J = 37.4Hz), 115.5 (q, J = 285.6 Hz), 83.4, 49.0, 43.5, 29.7, 27.6.

**HRMS (CI)**	Calculated	Found
$C_{11}H_{16}F_3NO_4$ [M+1]$^+$	284.1065	284.1080

***tert*-Butyl 5-chloro-4-oxo-2-(2,2,2-trifluoroacetamido)pentanoate (2b)**

According to the general procedure for Dess–Martin oxidations alcohol **1b** (422 mg, 1.32 mmol) and Dess–Martin periodinane (721 mg, 1.7 mmol) were reacted to

give **2b** after flash chromatography (silica, hexanes/EtOAc 8:2) in 90% yield (377 mg, 1.18 mmol) as a colorless solid with a melting point of 51 °C. [TLC: Hex/EA 75:25, R_f = 0.50]

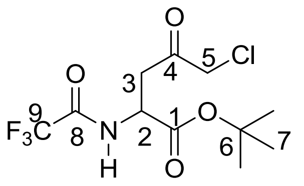

^{1}H-NMR (400 MHz, $CDCl_3$): δ = 7.29 (bs, 1 H, N-H), 4.69 (td, $^3J_{2,N-H}$ = 8.0 Hz, $^3J_{2,3}$ = 4.2 Hz, 1 H, 2-H), 3.24 (dd, $^2J_{3b,3a}$ = 18.6 Hz, $^3J_{3b,2}$ = 4.2 Hz, 1 H, 3b-H), 3.19 (dd, $^2J_{3a,3b}$ = 18.8 Hz, $^3J_{3a,2}$ = 4.4 Hz, 1 H, 3a-H), 2.17 (d, $^2J_{5a,5b}$ = $^2J_{5b,5a}$ = 2.8 Hz, 2 H, 5-H), 1.45 (s, 9 H, 7-H). **^{13}C-NMR** (100 MHz, $CDCl_3$): δ = 200.7, 167.7, 157.1 (q, *J* = 37.7 Hz), 118.3 (q, *J* = 285.8 Hz), 84.0, 49.0, 47.4, 40.5, 27.7.

**HRMS (CI):**	Calculated	Found
$C_{11}H_{15}ClF_3NO_4$ $[M]^+$	317.0642	317.0764

tert-Butyl 4-oxo-5-phenoxy-2-(2,2,2-trifluoroacetamido)pentanoate (2c)

According to the general procedure for Dess–Martin oxidations alcohol **1c** (498 mg, 1.32 mmol) and Dess–Martin periodinane (721 mg, 1.7 mmol) were reacted to give **11f** after flash chromatography (silica, hexanes/EtOAc 8:2) in 93% yield (461 mg, 1.23 mmol) as a colorless solid with a melting point of 58 °C. [TLC: Hex/EA 7:3, R_f = 0.55]

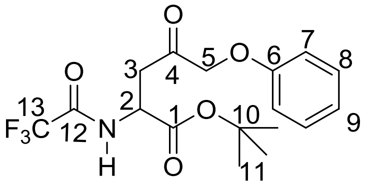

^{1}H-NMR (400 MHz, $CDCl_3$): δ = 7.29–7.33 (m, 2 H, 8-H), 7.02 (dd, $^3J_{9,8a}$ = $^3J_{9,8b}$ = 7.6 Hz, 1 H, 9-H), 6.86–6.88 (m, 2 H, 7-H), 4.73 (td, $^3J_{2,N-H}$ = 7.6 Hz, $^3J_{2,3}$ = 4.3 Hz, 1 H, 2-H), 4.57 (d, $^2J_{5a,5b}$ = $^2J_{5b,5a}$ = 1.2 Hz, 2 H, 5-H), 3.21 (dd, $^2J_{3a,3b}$ = 18.8 Hz, $^3J_{3a,2}$ = 4.4 Hz 1 H, 3a-H), 3.42 (dd, $^2J_{3b,3a}$ = 18.8 Hz, $^3J_{3b,2}$ = 4.4 Hz, 1 H, 3b-H), 1.45 (s, 9 H, 11-H). **^{13}C-NMR** (100 MHz, $CDCl_3$): δ = 205.9, 168.0, 157.3 157.2 (q, *J* = 37.4 Hz), 129.7, 122.1, 115.5 (q, *J* = 289.0 Hz), 114.4, 83.7, 72.5, 48.7, 40.4, 27.7.

**HRMS (CI)**	Calculated	Found
$C_{17}H_{20}F_3NO_5[M]^+$	375.1294	375.1289

Elemental Analysis:

$C_{17}H_{20}F_3NO_5$	Calculated	C	54.40	H	5.37	N	3.73
(375.34):	Found	C	54.73	H	5.75	N	3.79

***tert*-Butyl 5-(4-chlorophenoxy)-4-oxo-2-(2,2,2-trifluoroacetamido)pentanoate (2d)**

According to the general procedure for Dess–Martin oxidations alcohol **1d** (544 mg, 1.32 mmol) and Dess–Martin periodinane (721 mg, 1.7 mmol) were reacted to give **2d** after flash chromatography (silica, hexanes/EtOAc 8:2) in 82% yield (444 mg, 1.08 mmol) as a colorless oil. [TLC: Hex/EA 7:3, R_f = 0.51]

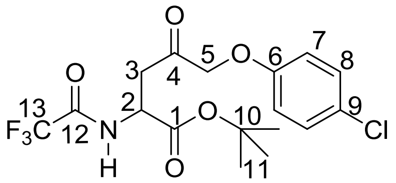

^{1}H-NMR (400 MHz, $CDCl_3$): δ = 7.32 (d, $^3J_{NH,2}$ = 7.3 Hz, 1 H, N-H), 7.22–7.25 (m, 2 H, 7-H), 6.77–6.81 (m, 2 H, 8-H), 4.71 (td, $^3J_{2,NH}$ = 8.0 Hz, $^3J_{2,3}$ = 4.2 Hz, 1 H, 2-H), 4.53 (d, $^2J_{5a,5b}$ = $^2J_{5b,5a}$ = 1.3 Hz, 2 H, 5-H), 3.36 (dd, $^2J_{3a,3b}$ = 18.8 Hz, $^3J_{3a,2}$ = 4.2 Hz, 1 H, 3a-H), 3.19 (dd, $^2J_{3b,3a}$ = 18.8 Hz, $^3J_{3b,2}$ = 4.2 Hz, 1 H, 3b-H), 1.43 (s, 9 H, 12-H). **^{13}C-NMR** (100 MHz, $CDCl_3$): δ = 205.1, 167.9, 157.2 (q, J = 37.4 Hz), 155.9, 129.6, 127.0, 115.7, 115.5 (q, J = 285.8 Hz), 114.0, 83.8, 72.6, 48.7, 40.3, 27.7.

**HRMS (CI):**	Calculated	Found
$C_{17}H_{19}ClF_3NO_5[M]^+$	409.0904	409.0883

***tert*-Butyl 5-(2-nitrophenoxy)-4-oxo-2-(2,2,2-trifluoroacetamido)pentanoate (2e)**

According to the general procedure for Dess–Martin oxidations alcohol **1e** (558 mg, 1.32 mmol) and Dess–Martin periodinane (721 mg, 1.7 mmol) were reacted to give **2e** after flash chromatography (silica, hexanes/EtOAc 8:2) in 87% yield (483 mg, 1.15 mmol) as a white solid with a melting point of 91 °C. [TLC: Hex/EA 8:2, R_f = 0.48]

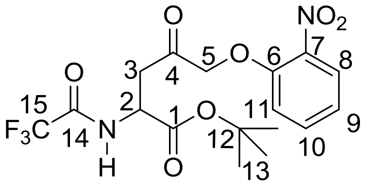

^{1}H-NMR (400 MHz, $CDCl_3$): δ = 7.93 (dd, $^3J_{8,9}$ = 8.1 Hz, $^3J_{8,10}$ = 1.7 Hz, 1 H, 8-H), 7.53–7.57 (m, 1 H, 9-H), 7.28 (bs, 1 H, N-H), 7.12–7.16 (m, 1 H, 10-H), 6.96 (dd, $^3J_{11,10}$ = 8.5 Hz, $^3J_{11,9}$ = 1.0 Hz, 1 H, 11-H), 4.79 (td, $^3J_{2,NH}$ = 7.8 Hz, $^3J_{2,3}$ = 4.3 Hz, 1 H, 2-H), 4.72 (d, $^2J_{5a,5b}$ = $^2J_{5b,5a}$ = 5.0 Hz, 2 H, 5-H), 3.50 (dd, $^2J_{3a,3b}$ = 18.8 Hz, $^3J_{3a,2}$ = 4.5 Hz, 1 H, 3a-H), 3.32 (dd, $^2J_{3b,3a}$ = 18.8 Hz, $^3J_{3b,2}$ = 4.2 Hz, 1 H, 3b-H), 1.46 (s, 9 H,

12-H). **^{13}C-NMR** (100 MHz, $CDCl_3$): δ = 204.2, 168.0, 157.2 (q, *J* = 37.4 Hz), 150.7, 134.4, 126.2, 122.0, 115.5 (q, *J* = 285.8 Hz), 114.6, 83.9, 73.3, 48.8, 40.6, 27.7.

**HRMS (CI):**	Calculated	Found
$C_{17}H_{19}F_3N_2O_7[M]^+$	420.1144	420.1156

tert-Butyl 5-(4-nitrophenoxy)-4-oxo-2-(2,2,2-trifluoroacetamido)pentanoate (2f)

According to the general procedure for Dess–Martin oxidations alcohol **1f** (558 mg, 1.32 mmol) and Dess–Martin periodinane (721 mg, 1.7 mmol) were reacted to give **2f** after flash chromatography (silica, hexanes/EtOAc 8:2) in 84% yield (466 mg, 1.11 mmol) as white solid with a melting point of 90 °C. [TLC: Hex/EA 7:3, R_f = 0.49]

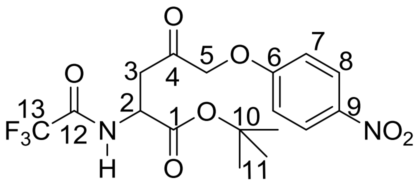

^{1}H-NMR (400 MHz, $CDCl_3$): δ = 8.21–8.27 (m, 2 H, 8-H), 7.35 (td, $^3J_{NH,2}$ = 6.5 Hz, 1 H, N-H), 6.95–6.99 (m, 2 H, 7-H), 4.75 (td, $^3J_{2,NH}$ = 7.3 Hz, $^3J_{2,3}$ = 4.5 Hz, 1 H, 2-H), 4.72 (d, $^2J_{5a,5b}$ = $^2J_{5b,5a}$ = 1.7 Hz, 2 H, 5-H), 3.37 (dd, $^2J_{3a,3b}$ = 18.5 Hz, $^3J_{3a,2}$ = 4.3 Hz, 1 H, 3a-H), 3.32 (dd, $^2J_{3b,3a}$ = 18.5 Hz, $^3J_{3b,2}$ = 4.4 Hz, 1 H, 3b-H), 1.47 (s, 9 H, 11-H). **^{13}C-NMR** (100 MHz, $CDCl_3$): δ = 205.1, 167.9, 157.2 (q, *J* = 37.4 Hz), 155.9, 129.6, 127.0, 115.7, 115.5 (q, *J* = 285.8 Hz), 114.0, 83.8, 72.6, 48.7, 40.3, 27.7.

**HRMS (CI):**	Calculated	Found
$C_{17}H_{19}F_3N_2O_7[M]^+$	420.1144	420.1171

General procedure for Passerini reactions

To a sample of pure γ-keto amino acid ester **2** or the aldehydes **5** and **7** (0.27 mmol) under N_2 in a 5 ml round bottom flask, acetic acid (17.6 mg, 0.29 mmol) and the corresponding isonitrile (0.29 mmol) were added. The resulting homogeneous solution was stirred at r.t. under nitrogen for 20 h. The crude product was purified by column chromatography (silica gel, EtOAc/hexane).

tert-Butyl 4-acetoxy-4-(chloromethyl)-5-(2-methoxy-2-oxoethylamino)-5-oxo-2-(2,2,2-trifluoroacetamido)pentanoate (3b)

According to the general procedure for Passerini reactions ketone **2b** (86 mg, 0.27 mmol), acetic acid (17.6 mg, 0.29 mmol) and methyl 2-isocyanoacetate (29 mg, 0.29 mmol) were reacted to give **3b** after flash chromatography (silica, hexanes/EtOAc 8:2) in 65% yield (84 mg, 0.18 mmol) as white solid with a melting point of 93 °C. [TLC: Hex/EA 7:3, R_f = 0.34]

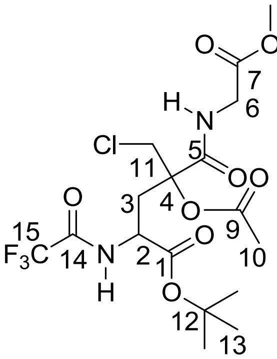

Major diastereomer:

^{1}H NMR (400 MHz, $CDCl_3$): δ = 7.50 (d, $^3J_{NHTFA,2}$ = 8.2 Hz, 1 H, N_{TFA}-H), 7.40 (t, $^3J_{NH,6}$ = 5.5 Hz, 1 H, N-H), 4.64–4.69 (m, 1 H, 2-H), 4.44 (d, $^2J_{11a,11b}$ = 11.6 Hz, 1 H, 11a-H), 4.31 (d, $^2J_{11b,11a}$ = 9.8 Hz, 1 H, 11b-H), 4.08 (dd, $^2J_{6a,6b}$ = 18.3 Hz, $^3J_{6a,2}$ = 5.27 Hz, 1 H, 6a-H), 3.92–3.98 (m, 1 H, 6b-H), 3.78 (s, 3 H, 8-H), 2.90 (dd, $^2J_{3a,3b}$ = 15.0 Hz, $^3J_{3a,2}$ = 10.4 Hz, 1 H, 3a-H), 2.53 (dd, $^2J_{3b,3a}$ = 14.7 Hz, $^3J_{3b,2}$ = 11.1 Hz, 1 H, 3b-H), 2.27 (s, 3 H, 10-H), 1.44 (s, 9 H, 13-H). **^{13}C NMR (100 MHz, $CDCl_3$):** δ = 170.1, 169.3, 168.8, 168.3, 157.0, 117.1, 83.9, 83.6, 52.6, 50.2, 45.7, 41.2, 34.8, 27.7, 21.6.

Minor diastereomer (selected signals)**:**

^{1}H NMR (400 MHz, $CDCl_3$): δ = 7.13 (t, $^3J_{NH,6}$ = 4.8 Hz, 1 H, N-H), 6.91 (d, $^3J_{NHTFA,2}$ = 8.7 Hz, 1 H, N_{TFA}-H), 4.44–4.50 (m, 1 H, 2-H), 4.16 (d, $^2J_{11a,11b}$ = 11.7 Hz, 1 H, 11a-H), 3.89–3.98 (m, 2 H, 6-H), 3.78 (s, 3 H, 8-H), 3.01 (dd, $^2J_{3a,3b}$ = 14.7 Hz, $^3J_{3a,2}$ = 3.3 Hz, 1 H, 3a-H), 2.43 (dd, $^2J_{3b,3a}$ = 15.0 Hz, $^3J_{3b,2}$ = 3.1 Hz, 1 H, 3b-H), 1.44 (s, 9 H, 13-H). **^{13}C NMR (100 MHz, $CDCl_3$):** δ = 169.4, 168.9, 168.8, 168.1, 83.8, 83.5, 49.1, 34.3, 21.3.

**HRMS (CI):**	Calculated	Found
$C_{17}H_{24}ClF_3N_2O_8$ $[M+1]^+$	477.1252	477.1209

tert-Butyl 4-acetoxy-5-(2-methoxy-2-oxoethylamino)-5-oxo-4-(phenoxymethyl)-2-(2,2,2-trifluoroacetamido)pentanoate (3c)

According to the general procedure for Passerini reactions ketone **2c** (101 mg, 0.27 mmol), acetic acid (17.6 mg, 0.29 mmol) and methyl 2-isocyanoacetate (29 mg, 0.29 mmol) were reacted to give **3c** after flash chromatography (silica, hexanes/EtOAc 8:2) in 57% yield (82 mg, 0.15 mmol) as a white solid with a melting point of 109 °C. [TLC: Hex/EA 7:3, R_f =0.35]

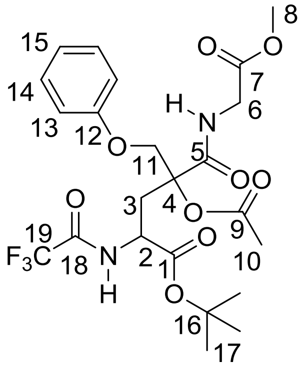

Major diastereomer:

^{1}H NMR (400 MHz, CDCl$_3$): δ = 7.61 (d, $^3J_{NHTFA,2}$ = 8.0 Hz, 1 H, N_{TFA}-H), 7.44 (t, $^3J_{NH,6}$ = 5.4 Hz, 1 H, N-H), 7.24–7.33 (m, 5 H, 13-H, 14-H, 15-H), 4.73 (d, $^2J_{11a,11b}$ = 9.8 Hz, 1 H, 11a-H), 4.54–4.59 (m, 1 H, 2-H), 4.43 (d, $^2J_{11b,11a}$ = 9.8 Hz, 1 H, 11b-H), 4.17–4.29 (m, 2 H, 6-H), 3.80 (s, 3 H, 8-H), 2.93 (dd, $^2J_{3a,3b}$ = 15.2 Hz, $^3J_{3a,2}$ = 9.9 Hz, 1 H, 3a-H), 2.65 (dd, $^2J_{3b,3a}$ = 14.7 Hz, $^3J_{3b,2}$ = 11.0 Hz, 1 H, 3b-H), 2.08 (s, 3 H, 10-H), 1.48 (s, 9 H, 17-H). **^{13}C NMR (100 MHz, CDCl$_3$):** δ = 170.1, 169.7, 169.1, 168.5, 156.7, 129.5, 121.8, 115.0, 114.9, 114.7, 83.5, 83.1, 68.5, 52.3, 50.1, 41.2, 34.2, 27.8, 21.5.

Minor diastereomer (selected signals)**:**

^{1}H NMR (400 MHz, CDCl$_3$): δ = 6.98–7.02 (m, 2 H, N-H, 15-H), 6.89–6.93 (m, 5 H, 13-H, 14-H, N_{TFA}-H), 4.88 (d, $^2J_{11a,11b}$ = 9.9 Hz, 1 H, 11a-H), 4.69–4.74 (m, 1 H, 2-H), 4.22 (d, $^2J_{11b,11a}$ = 9.7 Hz, 1 H, 11b-H), 3.92–4.04 (m, 2 H, 6-H), 3.79 (s, 3 H, 8-H), 2.53 2.65 (dd, $^2J_{3b,3a}$ = 15.2 Hz, $^3J_{3b,2}$ = 3.2 Hz, 1 H, 3b-H), 2.19 (s, 3 H, 10-H), 1.48 (s, 9 H, 17-H). **^{13}C NMR (100 MHz, CDCl$_3$):** δ = 170.1, 169.5, 169.1, 168.4, 129.5, 83.4, 83.0, 68.3, 48.9, 33.0, 27.8, 21.3.

**HRMS (CI):**	Calculated	Found
$C_{23}H_{29}F_3N_2O_9$ [M-C_4H_9]$^+$	477.1121	477.1175

***tert*-Butyl 4-acetoxy-4-[(4-chlorophenoxy)methyl]-5-(2-methoxy-2-oxoethyl-amino)-5-oxo-2-(2,2,2-trifluoroacetamido)pentanoate (3d)**

According to the general procedure for Passerini reactions ketone **2d** (111 mg, 0.27 mmol), acetic acid (17.6 mg, 0.29 mmol) and methyl 2-isocyanoacetate (29 mg, 0.29 mmol) were reacted to give **3d** after flash chromatography (silica, hexanes/EtOAc 8:2) in 69% yield (107 mg, 0.19 mmol) as a white solid with a melting point of 102 °C. [TLC: Hex/EA 7:3, R_f =0.33]

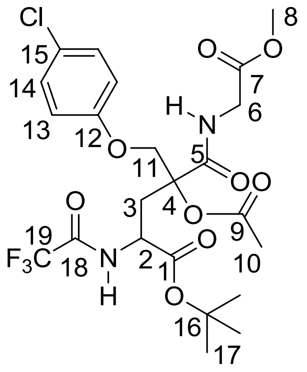

Major diastereomer:

^{1}H NMR (400 MHz, CDCl$_3$): δ = 7.56 (d, $^3J_{NHTFA,2}$ = 8.2 Hz, 1 H, N$_{TFA}$-H), 7.41 (t, $^3J_{NH,6}$ = 5.5 Hz, 1 H, N-H), 7.20–7.23 (m, 2 H, 14-H), 6.79–6.83 (m, 2 H, 13-H), 4.83 (d, $^2J_{11a,11b}$ = 9.8 Hz, 1 H, 11a-H), 4.50–4.53 (m, 1 H, 2-H), 4.41 (d, $^2J_{11b,11a}$ = 9.8 Hz, 1 H, 11b-H), 4.10–4.28 (m, 2 H, 6-H), 3.77 (s, 3 H, 8-H), 2.84 (dd, $^2J_{3a,3b}$ = 15.0 Hz, $^3J_{3a,2}$ = 10.0 Hz, 1 H, 3a-H), 2.60 (dd, $^2J_{3b,3a}$ = 14.7 Hz, $^3J_{3b,2}$ = 11.0 Hz, 1 H, 3b-H), 2.18 (s, 3 H, 10-H), 1.46 (s, 9 H, 17-H). **^{13}C NMR (100 MHz, CDCl$_3$):** δ = 170.1, 169.5, 169.1, 168.5, 156.4, 129.5, 129.4, 126.7, 116.2, 116.0, 83.6, 83.1, 68.9, 52.5, 50.0, 41.2, 34.1, 27.8, 21.6.

Minor diastereomer (selected signals)**:**

^{1}H NMR (400 MHz, CDCl$_3$): δ = 7.20–7.23 (m, 3 H, 14-H, N-H), 6.93 (d, $^3J_{NHTFA,2}$ = 9.0 Hz, 1 H, N$_{TFA}$-H), 6.80–6.83 (m, 2 H, 13-H), 4.66 (d, $^2J_{11a,11b}$ = 9.8 Hz, 1 H, 11a-H), 4.66–4.72 (m, 1 H, 2-H), 4.17 (d, $^2J_{11b,11a}$ = 9.8 Hz, 1 H, 11b-H), 3.87–4.00 (m, 2 H, 6-H), 3.77 (s, 3 H, 8-H), 2.97 (dd, $^2J_{3a,3b}$ = 14.7 Hz, $^3J_{3a,2}$ = 3.3 Hz, 1 H, 3a-H), 2.50 (dd, $^2J_{3b,3a}$ = 15.0 Hz, $^3J_{3b,2}$ = 3.2 Hz, 1 H, 3b-H), 2.06 (s, 3 H, 10-H), 1.45 (s, 9 H, 17-H). **^{13}C NMR (100 MHz, CDCl$_3$):** δ = 170.0, 169.5, 168.4, 129.4, 116.2, 83.5, 83.1, 68.6, 48.8, 41.1, 32.9, 27.8, 21.3.

**HRMS (CI):**	Calculated	Found
$C_{23}H_{28}{}^{37}ClF_3N_2O_9$ [M]$^+$	570.1400	570.1473

tert-Butyl 4-acetoxy-5-(2-methoxy-2-oxoethylamino)-4-[(2-nitrophenoxy)-methyl]-5-oxo-2-(2,2,2-trifluoroacetamido)pentanoate (3e)

According to the general procedure for Passerini reactions ketone **2e** (113 mg, 0.27 mmol), acetic acid (17.6 mg, 0.29 mmol) and methyl 2-isocyanoacetate (29 mg, 0.29 mmol) were reacted to give **3e** after flash chromatography (silica, hexanes/ EtOAc 8:2) in 62% yield (97 mg, 0.17 mmol) as a white solid with a melting point of 105 °C. [TLC: Hex/EA 7:3, R_f =0.33]

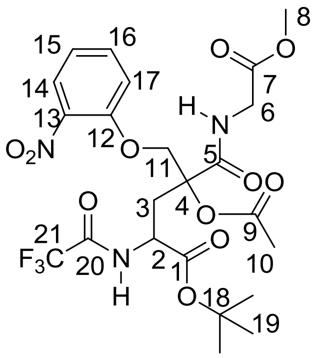

Major diastereomer:

^{1}H NMR (400 MHz, $CDCl_3$): δ = 7.84–7.88 (m, 1 H, 14-H), 7.78 (d, $^3J_{NHTFA,2}$ = 8.0 Hz, 1 H, N_{TFA}-H), 7.45 (t, $^3J_{NH,6}$ = 5.8 Hz, 1 H, N-H), 7.51–7.55 (m, 1 H, 17-H), 6.99–7.09 (m, 2 H, 15-H, 16-H), 4.93 (d, $^2J_{11a,11b}$ = 9.3 Hz, 1 H, 11a-H), 4.57–4.73 (m, 1 H, 2-H), 4.53 (d, $^2J_{11b,11a}$ = 9.3 Hz, 1 H, 11b-H), 4.28 (dd, $^2J_{6a,6b}$ = 17.9 Hz, $^3J_{6a,NH}$ = 6.7 Hz, 1 H, 6a-H), 3.29 (dd, $^2J_{6b,6a}$ = 17.9 Hz, $^3J_{6b,NH}$ = 5.1 Hz, 1 H, 6b-H), 3.76 (s, 3 H, 8-H), 2.65 (dd, $^2J_{3a,3b}$ = 14.7 Hz, $^3J_{3a,2}$ = 11.0 Hz, 1 H, 3a-H), 2.55 (dd, $^2J_{3b,3a}$ = 15.0 Hz, $^3J_{3b,2}$ = 3.5 Hz, 1 H, 3b-H), 2.10 (s, 3 H, 10-H), 1.45 (s, 9 H, 19-H). **^{13}C NMR (100 MHz, $CDCl_3$):** δ = 170.2, 169.6, 169.4, 168.3, 151.0, 139.5, 134.5, 125.9, 121.3, 117.0, 114.6, 83.4, 82.6, 69.4, 52.5, 50.2, 48.8, 41.3, 33.2, 27.7, 21.3.

Minor diastereomer (selected signals)**:**

^{1}H NMR (400 MHz, $CDCl_3$): δ = 7.32 (t, $^3J_{NH,6}$ = 5.4 Hz, 1 H, N-H), 6.99-7.09 (m, 2 H, 15-H, 16-H), 5.08 (d, $^2J_{11a,11b}$ = 9.3 Hz, 1 H, 11a-H), 4.50–4.56 (m, 1 H, 2-H), 4.36 (d, $^2J_{11b,11a}$ = 9.3 Hz, 1 H, 11b-H), 4.01–4.03 (m, 2 H, 6-H), 3.72 (s, 3 H, 8-H), 2.96-3.02 (m, 2 H, 3-H), 2.23 (s, 3 H, 10-H), 1.45 (s, 9 H, 19-H). **^{13}C NMR (100 MHz, $CDCl_3$):** δ = 169.7, 169.5, 169.3, 168.2, 151.1, 139.4, 125.6, 121.3, 114.4, 82.4, 69.5, 52.4, 41.2, 32.5, 21.5.

**HRMS (CI):**	Calculated	Found
$C_{23}H_{28}F_3N_3O_{11}$ $[M+1]^+$	580.1709	580.1798

***tert*-Butyl 4-acetoxy-5-(2-ethoxy-2-oxoethylamino)-4-[(4-nitrophenoxy)methyl]-5-oxo-2-(2,2,2-trifluoroacetamido)pentanoate (3f)**

According to the general procedure for Passerini reactions ketone **2f** (111 mg, 0.27 mmol), acetic acid (17.6 mg, 0.29 mmol) and ethyl 2-isocyanoacetate (33 mg, 0.29 mmol) were reacted to give **3f** after flash chromatography (silica, hexanes/ EtOAc 8:2) in 69% yield (108 mg, 0.19 mmol) as a white solid with a melting point of 108 °C. [TLC: Hex/EA 7:3, R_f = 0.34]

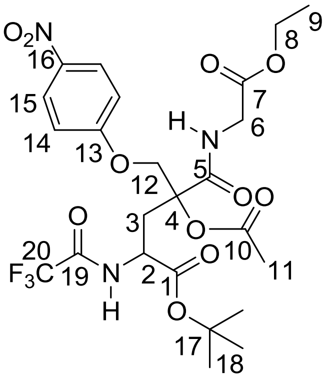

Major diastereomer:

^{1}H NMR (400 MHz, $CDCl_3$): δ = 7.65 (d, $^{3}J_{NHTFA,2}$ = 8.0 Hz, 1 H, N_{TFA}-H), 7.39 (t, $^{3}J_{NH,6}$ = 5.4 Hz, 1 H, N-H), 7.18–7.23 (m, 2 H, 15-H), 6.78–6.82 (m, 2 H, 14-H), 4.83 (d, $^{2}J_{11a,11b}$ = 9.9 Hz, 1 H, 11a-H), 4.50–4.55 (m, 1 H, 2-H), 4.40 (d, $^{2}J_{11b,11a}$ = 9.8 Hz, 1 H, 11b-H), 4.16–4.26 (m, 4 H, 6-H, 8-H), 2.90 (dd, $^{2}J_{3a,3b}$ = 15.0 Hz, $^{3}J_{3a,2}$ = 10.0 Hz, 1 H, 3a-H), 2.60 (dd, $^{2}J_{3b,3a}$ = 14.7 Hz, $^{3}J_{3b,2}$ = 11.2 Hz, 1 H, 3b-H), 2.18 (s, 3 H, 11-H), 1.45 (s, 9 H, 18-H), 1.28 (t, $^{3}J_{9,8}$ = 7.2 Hz, 3 H, 9-H). **^{13}C NMR (100 MHz, $CDCl_3$):** δ = 169.9, 169.5, 169.1, 168.5, 156.7, 156.4, 129.4, 126.7, 116.3, 115.5, 83.5, 83.1, 68.9, 61.8, 50.1, 41.3, 33.9, 27.8, 21.6, 14.0.

Minor diastereomer (selected signals)**:**

^{1}H NMR (400 MHz, $CDCl_3$): δ = 7.02 (d, $^{3}J_{NHTFA,2}$ = 8.9 Hz, 1 H, N_{TFA}-H), 6.79–6.83 (m, 2 H, 14-H), 4.67 (d, $^{2}J_{11a,11b}$ = 9.8 Hz, 1 H, 11a-H), 4.64–4.67 (m, 1 H, 2-H), 2.97 (dd, $^{2}J_{3a,3b}$ = 14.7 Hz, $^{3}J_{3a,2}$ = 3.3 Hz, 1 H, 3a-H), 2.50 (dd, $^{2}J_{3b,3a}$ = 15.0 Hz, $^{3}J_{3b,2}$ = 3.3 Hz, 1 H, 3b-H), 2.06 (s, 3 H, 11-H), 1.45 (s, 9 H, 18-H), 1.28 (t, $^{3}J_{9,8}$ = 7.2 Hz, 3 H, 9-H). **^{13}C NMR (100 MHz, $CDCl_3$):** δ = 169.7, 169.1, 169.1, 168.4, 116.3, 83.4, 83.1, 68.6, 48.8, 41.3, 32.8, 21.3.

**HRMS (CI):**	Calculated	Found
$C_{24}H_{30}F_3N_3O_{11}$ $[M+1]^+$	594.1866	594.1933

tert-Butyl 4-acetoxy-4-[(4-chlorophenoxy)methyl]-5-(2-ethoxy-2-oxoethylamino)-5-oxo-2-(2,2,2-trifluoroacetamido)pentanoate (3g)

According to the general procedure for Passerini reactions ketone **2d** (111 mg, 0.27 mmol), acetic acid (17.6 mg, 0.29 mmol) and ethyl 2-isocyanoacetate (33 mg, 0.29 mmol) were reacted to give **3g** after flash chromatography (silica, hexanes/ EtOAc 8:2) in 68% yield (108 mg, 0.19 mmol) as a white solid with a melting point of 107 °C. [TLC: Hex/EA 7:3, R_f = 0.33]

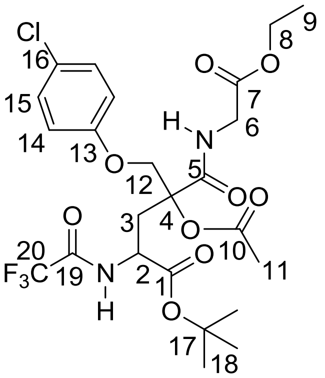

Major diastereomer:

^{1}H NMR (400 MHz, CDCl$_3$): δ = 7.64 (d, $^3J_{NHTFA,2}$ = 8.0 Hz, 1 H, N$_{TFA}$-H), 7.38 (t, $^3J_{NH,6}$ = 5.5 Hz, 1 H, N-H), 7.18–7.23 (m, 2 H, 15-H), 6.78–6.82 (m, 2 H, 14-H), 4.83 (d, $^2J_{11a,11b}$ = 9.9 Hz, 1 H, 11a-H), 4.50–4.55 (m, 1 H, 2-H), 4.40 (d, $^2J_{11b,11a}$ = 9.8 Hz, 1 H, 11b-H), 4.16–4.26 (m, 4 H, 6-H, 8-H), 2.90 (dd, $^2J_{3a,3b}$ = 15.0 Hz, $^3J_{3a,2}$ = 10.0 Hz, 1 H, 3a-H), 2.60 (dd, $^2J_{3b,3a}$ = 14.7 Hz, $^3J_{3b,2}$ = 11.2 Hz, 1 H, 3b-H), 2.18 (s, 3 H, 11-H), 1.45 (s, 9 H, 18-H), 1.28 (t, $^3J_{9,8}$ = 7.2 Hz, 3 H, 9-H). **^{13}C NMR (100 MHz, CDCl$_3$):** δ = 169.9, 169.5, 169.1, 168.5, 156.7, 156.4, 129.4, 126.7, 116.3, 115.5, 83.5, 83.1, 68.9, 61.8, 50.1, 41.3, 33.9, 27.8, 21.6, 14.0.

Minor diastereomer (selected signals)**:**

^{1}H NMR (400 MHz, CDCl$_3$): δ = 7.19–7.23 (m, 3 H, N-H, 15-H), 7.02 (d, $^3J_{NHTFA,2}$ = 8.9 Hz, 1 H, N$_{TFA}$-H), 6.79–6.83 (m, 2 H, 14-H), 4.67 (d, $^2J_{11a,11b}$ = 9.8 Hz, 1 H, 11a-H), 4.64–4.67 (m, 1 H, 2-H), 2.97 (dd, $^2J_{3a,3b}$ = 14.7 Hz, $^3J_{3a,2}$ = 3.3 Hz, 1 H, 3a-H), 2.50 (dd, $^2J_{3b,3a}$ = 15.0 Hz, $^3J_{3b,2}$ = 3.3 Hz, 1 H, 3b-H), 2.06 (s, 3 H, 11-H), 1.45 (s, 9 H, 18-H), 1.28 (t, $^3J_{9,8}$ = 7.2 Hz, 3 H, 9-H). **^{13}C NMR (100 MHz, CDCl$_3$):** δ = 169.7, 169.1, 169.1, 168.4, 116.3, 83.4, 83.1, 68.6, 48.8, 41.3, 32.8, 21.3.

**HRMS (CI):**	Calculated	Found
$C_{24}H_{30}ClF_3N_2O_9$ [M]$^+$	582.1592	582.1563

(*E*)-*tert*-Butyl 6-oxo-2-(2,2,2-trifluoroacetamido)hex-4-enoate (5)

According to the general procedure for Dess–Martin oxidations alcohol **4**[2] (595 mg, 2.00 mmol) and Dess–Martin periodinane (1.10 g, 2.60 mmol) were reacted to give **5** after flash chromatography (silica, hexanes/EtOAc 9:1, 8:2, 7:3) in 79% yield (467 mg, 1.58 mmol) as a pale yellow oil. [TLC: Hex/EA 1/1, R_f = 0.78]

[2] Thies, S., Kazmaier U. *Synlett* **2010**, 137–141.

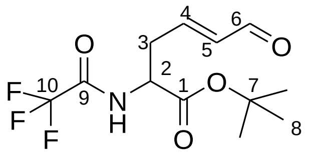

^{1}H NMR (400 MHz, $CDCl_3$): δ = 9.51 (d, $^3J_{6,5}$ = 7.7 Hz, 1H, 6-H), 7.02 (m, 1H, NH), 6.67 (dt, $^3J_{4,5}$ = 15.6 Hz, $^3J_{4,3}$ = 7.4 Hz, 1H, 4-H), 5.69 (ddt, $^3J_{5,4}$ = 15.6 Hz, $^3J_{5,6}$ = 7.7 Hz, $^4J_{5,3}$ = 1.3 Hz, 1H, 5-H), 4.58 (td, $^3J_{2,3}$ = $^3J_{2,NH}$ = 5.9 Hz, 1H, 2-H), 2.99 (dddd, $^2J_{3a,3b}$ = 14.9 Hz, $^3J_{3a,4}$ = 7.4 Hz, $^3J_{3a,2}$ = 5.8 Hz, $^4J_{3a,5}$ = 1.3 Hz, 1H, 3-Ha), 2.82 (dddd, $^2J_{3b,3a}$ = 14.9 Hz, $^3J_{3b,4}$ = 7.4 Hz, $^3J_{3b,2}$ = 5.8 Hz, $^4J_{3b,5}$ = 1.4 Hz, 1H, 3-Hb), 1.49 (s, 9H, 8-H). **^{13}C NMR (100 MHz, $CDCl_3$):** δ = 192.8, 168.5, 149.2, 136.1, 84.6, 52.0, 35.0, 28.0, (C-9, C-10 could not be detected).

**HRMS (ESI):**	Calculated	Found
$C_{12}H_{16}F_3NNaO_4$ $[M+Na]^+$	318.0929	318.0925

(*E*)-*tert*-Butyl 6-acetoxy-7-(2-ethoxy-2-oxoethylamino)-7-oxo-2-(2,2,2-trifluoro-acetamido)hept-4-enoate (6a)

According to the general procedure for Passerini reactions aldehyde **5** (44 mg, 0.15 mmol), acetic acid (10 mg, 0.17 mmol) and ethyl 2-isocyanoacetate (19 mg, 0.17 mmol) were reacted to give **6a** after flash chromatography (silica, hexanes/ EtOAc 6/4, 1/1) in 64% yield (45 mg, 0.096 mmol) as a colorless oil. [TLC: Hex/EA 1/1, R_f = 0.36]

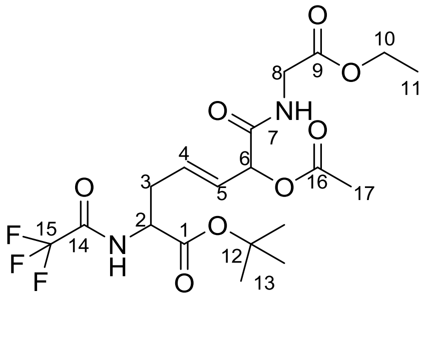

Diastereomer 1:

^{1}H NMR (400 MHz, $CDCl_3$): δ = 7.15 (d, $^3J_{NH,2}$ = 7.3 Hz, 1H, NH), 6.63 (d, $^3J_{NH,8}$ = 5.1 Hz, 1H, NH), 5.82, 5.79 (2dt, $^3J_{4,5}$ = 15.3 Hz, $^3J_{4,3}$ = 7.4 Hz, 1H, 4-H), 5.72, 5.68 (2dd, $^3J_{5,4}$ = 15.3 Hz, $^3J_{5,6}$ = 6.7 Hz, 1H, 5-H), 5.59 (d, $^3J_{6,5}$ = 6.7 Hz, 1H, 6-H), 4.60, 4.56 (2dt, $^3J_{2,NH}$ = 7.3 Hz, $^3J_{2,3}$ = 5.6 Hz, 1H, 2-H), 4.24 (q, $^3J_{10,11}$ = 7.1 Hz, 2H, 10-H), 4.09 (dd, $^2J_{8a,8b}$ = 18.3 Hz, $^3J_{8a,NH}$ = 5.5 Hz, 1H, 8-Ha), 4.00 (dd, $^2J_{8b,8a}$ = 18.3 Hz, $^3J_{8b,NH}$ = 5.0 Hz, 1H, 8-Hb), 2.71 (m, 1H, 3-Ha), 2.60 (m, 1H, 3-Hb), 2.18 (s, 3H, 17-H), 1.49

(s, 9H, 13-H), 1.31 (t, $^3J_{11,10}$ = 7.1 Hz, 3H, 11-H). **^{13}C NMR (100 MHz, CDCl$_3$):** δ = 169.5, 169.0, 168.9, 167.9, 156.7 ($J_{14,F}$ = 37.9 Hz), 129.5, 128.5, 115.6 ($J_{15,F}$ = 287.6 Hz), 83.7, 73.6, 61.7, 52.4, 41.0, 34.6, 27.9, 20.8, 14.0.

Diastereomer 2: (selected signals)**:**

^{1}H NMR (400 MHz, CDCl$_3$): δ = 7.08 (d, $^3J_{NH,2}$ = 7.2 Hz, NH), 6.59 (d, $^3J_{NH,8}$ = 5.1 Hz, 1H, NH), 5.57 (d, $^3J_{6,5}$ = 6.7 Hz, 1H, 6-H), 4.07 (dd, $^2J_{8a,8b}$ = 18.3 Hz, $^3J_{8a,NH}$ = 5.3 Hz, 1H, 8-Ha), 3.99 (dd, $^2J_{8b,8a}$ = 18.3 Hz, $^3J_{8b,NH}$ = 4.7 Hz, 1H, 8-Hb), 2.17 (s, 3H, 17-H). **^{13}C NMR (100 MHz, CDCl$_3$):** δ = 169.5, 169.0, 156.6 ($J_{14,F}$ = 37.9 Hz), 129.3, 128.4, 83.6, 73.4, 52.4, 34.5.

**HRMS (CI)**:	Calculated	Found
$C_{17}H_{24}F_3N_2O_6$ [M-$C_2H_3O_2$]$^+$	409.1586	409.1628

(*E*)-7-*tert*-Butoxy-1-(2-ethoxy-2-oxoethylamino)-1,7-dioxo-6-(2,2,2-trifluoro-acetamido)hept-3-en-2-yl benzoate (6b)

According to the general procedure for Passerini reactions aldehyde **5** (44 mg, 0.15 mmol), benzoic acid (21 mg, 0.17 mmol) and ethyl 2-isocyanoacetate (19 mg, 0.17 mmol) were reacted to give **6b** after flash chromatography (silica, hexanes/ EtOAc 6/4) in 44% yield (35 mg, 0.066 mmol) as a colorless oil. [TLC: Hex/EA 1/1, R_f = 0.45]

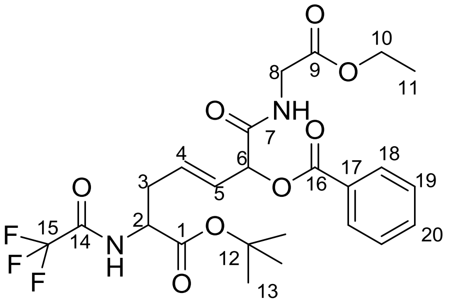

Diastereomer 1:

^{1}H NMR (400 MHz, CDCl$_3$): δ = 8.05 (m, 2H, 18-H), 7.60 (m, 1H, 20-H), 8.05 (m, 2H, 19-H), 7.13 (d, $^3J_{NH,2}$ = 7.0 Hz, 1H, NH), 6.72, 6.58 (2d, $^3J_{NH,8}$ = 5.0 Hz, 1H, NH), 5.87 (m, 3H, 4-H, 5-H, 6-H), 4.62, 4.59 (2dt, $^3J_{2,NH}$ = 7.0 Hz, $^3J_{2,3}$ = 5.6 Hz, 1H, 2-H), 4.22 (q, $^3J_{10,11}$ = 7.1 Hz, 2H, 10-H), 4.10 (m, 1H, 8-Ha), 4.06 (m, 1H, 8-Hb), 2.73 (m, 1H, 3-Ha), 2.61 (m, 1H, 3-Hb), 1.46 (s, 9H, 13-H), 1.27 (t, $^3J_{11,10}$ = 7.1 Hz, 3H, 11-H). **^{13}C NMR (100 MHz, CDCl$_3$):** δ = 169.4, 168.9, 168.0, 164.8, 157.0 ($J_{14,F}$ = 37.9 Hz), 133.7, 129.8, 129.2, 129.0, 128.6, 128.5, 83.7, 74.0, 61.7, 52.4, 41.1, 34.6, 27.9, 14.1, C-15 could not be detected.

Diastereomer 2 (selected signals)**:**

^{1}H NMR (400 MHz, CDCl$_3$): δ = 4.14 (dd, $^2J_{8a,8b}$ = 18.3 Hz, $^3J_{8a,NH}$ = 5.5 Hz, 1H, 8-Ha), 4.02 (dd, $^2J_{8b,8a}$ = 18.3 Hz, $^3J_{8b,NH}$ = 5.0 Hz, 1H, 8-Hb), 1.44 (s, 9H, 13-H). **^{13}C NMR (100 MHz, CDCl$_3$):** δ = 169.4, 168.8, 168.0, 129.8, 129.1, 128.9, 128.4 83.7, 73.8, 34.5.

**HRMS (CI):**	Calculated	Found
$C_{20}H_{21}F_3N_2O_8$ [M-C_4H_8]$^+$	474.1250	474.1206

(*E*)-*tert*-Butyl 6-(2-(benzyloxycarbonylamino)acetoxy)-7-(2-ethoxy-2-oxoethyl-amino)-7-oxo-2-(2,2,2-trifluoroacetamido)hept-4-enoate (6c)

According to the general procedure for Passerini reactions aldehyde **5** (44 mg, 0.15 mmol), Cbz-glycine (36 mg, 0.17 mmol) and ethyl 2-isocyanoacetate (19 mg, 0.17 mmol) were reacted to give **6c** after flash chromatography (silica, hexanes/EtOAc 6/4, 1/1) in 43% yield (37 mg, 0.064 mmol) as a colorless oil. [TLC: Hex/EA 1/1, R_f = 0.26]

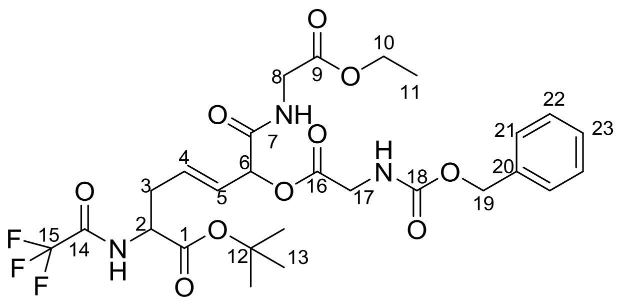

Diastereomer 1:

^{1}H NMR (400 MHz, CDCl$_3$): δ = 7.37 (m, 5H, 21-H, 22-H, 23-H), 7.27 (d, $^3J_{NH,2}$ = 8.3 Hz, 1H, NH), 6.86 (m, 1H, NH), 5.85 (dt, $^3J_{4,5}$ = 15.1 Hz, $^3J_{4,3}$ = 7.9 Hz, 1H, 4-H), 5.75, 5.70 (2dd, $^3J_{5,4}$ = 15.1 Hz, $^3J_{5,6}$ = 6.2 Hz, 1H, 5-H), 5.66, 5.64 (2d, $^3J_{6,5}$ = 6.2 Hz, 1H, 6-H), 5.46 (m, 1H, NH), 5.14 (m, 2H, 19-H), 4.59 (m, 1H, 2-H), 4.21 (q, $^3J_{10,11}$ = 7.1 Hz, 2H, 10-H), 3.98 (m, 4H, 8-H, 17-H), 2.70 (ddd, $^2J_{3a,3b}$ = 14.4 Hz, $^3J_{3a,4}$ = 8.6 Hz, $^3J_{3a,2}$ = 5.5 Hz, 1H, 3-Ha), 2.56 (ddd, $^2J_{3b,3a}$ = 14.4 Hz, $^3J_{3b,4}$ = $^3J_{3b,2}$ = 6.9 Hz, 1H, 3-Hb), 1.49 (s, 9H, 13-H), 1.29 (t, $^3J_{11,10}$ = 7.1 Hz, 3H, 11-H). **^{13}C NMR (100 MHz, CDCl$_3$):** δ = 169.4, 168.9, 168.5, 167.7, 156.7 ($J_{14,F}$ = 37.3 Hz), 136.1, 130.4, 128.5, 128.3, 128.1, 127.8, 115.5 ($J_{15,F}$ = 288.3 Hz), 83.7, 74.1, 67.3, 61.6, 52.5, 42.9, 41.1, 34.7, 27.9, 14.1.

Diastereomer 2 (selected signals)**:**

^{13}C NMR (100 MHz, CDCl$_3$): δ = 169.4, 168.8, 168.4, 129.8, 127.6.

**HRMS (CI):**	Calculated	Found
$C_{23}H_{26}F_3N_3O_{10}$ [M-C_4H_8]$^+$	561.1570	561.1561

tert-Butyl 6-oxo-2-(2,2,2-trifluoroacetamido)hexanoate (7)

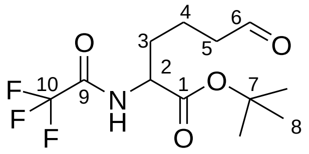

^{1}H NMR (400 MHz, CDCl$_3$): δ = 9.76 (t, $^3J_{6,5}$ = 1.1 Hz, 1H, 6-H), 6.98 (m, 1H, NH), 4.47 (td, $^3J_{2,3}$ = $^3J_{2,NH}$ = 5.8 Hz, 1H, 2-H), 2.53 (td, $^3J_{5a,4}$ = 7.0 Hz, $^3J_{5a,6}$ = 1.1 Hz, 1H, 5-Ha), 2.52 (td, $^3J_{5b,4}$ = 7.0 Hz, $^3J_{5b,6}$ = 1.1 Hz, 1H, 5-Hb), 1.94 (m, 1H, 3-Ha), 1.80 (m, 1H, 3-Hb), 1.65 (m, 2H, 4-H), 1.50 (s, 9H, 8-H). **^{13}C NMR (100 MHz, CDCl$_3$):** δ = 201.2, 169.7, 156.8 ($J_{9,F}$ = 37.4 Hz), 115.6 ($J_{10,F}$ = 287.7 Hz), 83.7, 52.9, 42.9, 31.2, 27.9, 17.2.

**HRMS (ESI):**	Calculated	Found
$C_{12}H_{18}F_3NNaO_4$ [M+Na]$^+$:	320.1086	320.1080

tert-Butyl 6-acetoxy-7-(2-ethoxy-2-oxoethylamino)-7-oxo-2-(2,2,2-trifluoroacet-amido)heptanoate (8)

According to the general procedure for Passerini reactions aldehyde **7** (37 mg, 0.12 mmol), acetic acid (8 mg, 0.13 mmol) and ethyl 2-isocyanoacetate (15 mg, 0.13 mmol) were reacted to give **8** after flash chromatography (silica, hexanes/ EtOAc 6/4, 1/1) in 80% yield (47 mg, 0.099 mmol) as colourless oil. [TLC: Hex/EA 1/1, R_f = 0.31]

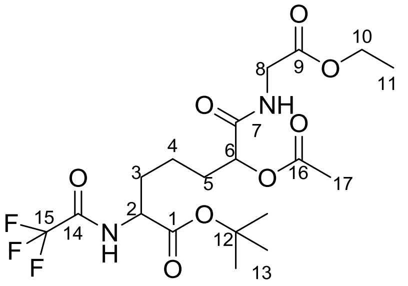

Diastereomer 1:

^{1}H NMR (400 MHz, CDCl$_3$): δ = 7.08, 7.05 (2d, $^3J_{NH,2}$ = 8.9 Hz, 1H, NH), 6.59 (t, $^3J_{NH,8}$ = 4.8 Hz, 1H, NH), 5.23 (t, $^3J_{6,5}$ = 5.8 Hz, 1H, 6-H), 4.45 (ddd, $^3J_{2,NH}$ = $^3J_{2,3a}$ = 7.3 Hz, $^3J_{2,3b}$ = 5.6 Hz, 1H, 2-H), 4.23 (q, $^3J_{10,11}$ = 7.1 Hz, 2H, 10-H), 4.07 (dd, $^2J_{8a,8b}$ = 18.3 Hz, $^3J_{8a,NH}$ = 5.3 Hz, 1H, 8-Ha), 3.99 (dd, $^2J_{8b,8a}$ = 18.3 Hz, $^3J_{8b,NH}$ = 2.6 Hz, 1H, 8-Hb), 2.16 (s, 3H, 17-H), 1.93 (m, 3H, 3-Ha, 5-H), 1.77 (m, 1H, 3-Hb), 1.48 (m, 11H, 4-H, 13-H), 1.29 (t, $^3J_{11,10}$ = 7.1 Hz, 3H, 11-H). **^{13}C NMR (100 MHz, CDCl$_3$):** δ = 169.8, 169.7, 169.6, 169.5, 156.8 ($J_{14,F}$ = 37.2 Hz), 115.9 ($J_{15,F}$ = 288.2 Hz), 83.3, 73.1, 61.7, 52.9, 41.0, 31.4, 31.2, 27.9, 20.8, 20.2, 14.1.

Diastereomer 2 (selected signals):

^{1}H NMR (400 MHz, CDCl$_3$): δ = 4.06 (dd, $^{2}J_{8a,8b}$ = 18.3 Hz, $^{3}J_{8a,NH}$ = 5.3 Hz, 1H, 8-Ha), 3.97 (dd, $^{2}J_{8b,8a}$ = 18.3 Hz, $^{3}J_{8b,NH}$ = 2.6 Hz, 1H, 8-Hb). **^{13}C NMR (100 MHz, CDCl$_3$):** δ = 169.5, 156.8 ($J_{14,F}$ = 37.5 Hz), 83.3, 52.9, 40.9, 31.3, 30.9, 27.9, 20.8, 20.0.

**HRMS (CI):**	Calculated	Found
$C_{15}H_{20}F_3N_2O_7$ [M-C_4H_9O]$^+$	397.1223	397.1190

***tert*-Butyl 6-(*N*-benzylacetamido)-7-(2-ethoxy-2-oxoethylamino)-7-oxo-2-(2,2,2-trifluoroacetamido)heptanoate (9)**

Aldehyde **7** (37 mg, 0.12 mmol) and benzylamine (15 mg, 0.13 mmol) were stirred for 15 min at 0 °C. Acetic acid (8 mg, 0.13 mmol) and ethyl 2-isocyanoacetate (15 mg, 0.13 mmol) were added and the mixture was allowed to warm up to r.t. overnight. The solvent was evaporated to give **9** after flash chromatography (silica, hexanes/ EtOAc 7/3, 1/1) in 34% yield (24 mg, 0.043 mmol) as a colorless oil. [TLC: Hex/EA 1/1, R_f = 0.43]

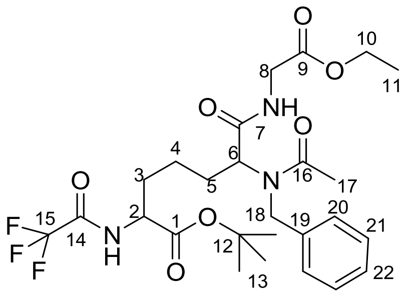

Diastereomer 1:

^{1}H NMR (400 MHz, CDCl$_3$): δ = 7.35 (m, 3H, 21-H, 22-H), 7.20 (d, $^{3}J_{20,21}$ = 7.4 Hz, 2H, 20-H), 7.11, 7.09 (2d, $^{3}J_{NH,2}$ = 8.1 Hz, 1H, NH), 7.00 (t, $^{3}J_{NH,8}$ = 5.7 Hz, 1H, NH), 5.00 (m, 1H, 6-H), 4.61 (s, 2H, 18-H), 4.40 (m, 1H, 2-H), 4.21 (q, $^{3}J_{10,11}$ = 7.0 Hz, 2H, 10-H), 3.94 (dd, $^{2}J_{8a,8b}$ = 18.1 Hz, $^{3}J_{8a,NH}$ = 6.2 Hz, 1H, 8-Ha), 3.89 (m, 1H, 8-Hb), 2.14 (s, 3H, 17-H), 1.96, 1.82, 1.70, 1.59 (m, 4H, 3-H, 5-H), 1.48 (s, 9H, 13-H), 1.29 (m, 5H, 4-H, 11-H). **^{13}C NMR (100 MHz, CDCl$_3$):** δ = 173.4, 170.7, 169.7, 169.7, 156.8 ($J_{14,F}$ = 37.7 Hz), 137.1, 128.8, 127.4, 126.0, 115.7 ($J_{15,F}$ = 288.0 Hz), 83.2, 61.4, 57.1, 53.1, 49.4, 41.0, 31.8, 27.7, 27.9, 22.3, 22.0, 14.1.

Diastereomer 2 (selected signals)**:**

^{1}H NMR (400 MHz, CDCl$_3$): δ = 6.94 (t, $^{3}J_{NH,8}$ = 5.6 Hz, NH), 4.60 (s, 2H, 18-H), 3.95 (dd, $^{2}J_{8a,8b}$ = 18.1 Hz, $^{3}J_{8a,NH}$ = 6.1 Hz, 1H, 8-Ha), 3.85 (dd, $^{2}J_{8b,8a}$ = 18.1 Hz, $^{3}J_{8b,NH}$ = 5.3 Hz, 1H, 8-Hb). **^{13}C NMR (100 MHz, CDCl$_3$):** δ = 83.1, 57.0, 52.9, 49.2, 31.3, 27.5, 21.7.

**HRMS (CI):**	Calculated	Found
$C_{26}H_{36}F_3N_3O_7$ [M]$^+$	559.2505	559.2480
